# Exacerbation of *N*-nitrosodiethylamine Induced Hepatotoxicity and DNA Damage in Mice Exposed to Chronic Unpredictable Stress

**DOI:** 10.3389/fphar.2017.00360

**Published:** 2017-06-15

**Authors:** Nayeem Bilal, Nida Suhail, Shirin Hasan, Ghulam M. Ashraf, Sabiha Fatima, Husain Y. Khan, Mariam S. Alharbi, Athanasios Alexiou, Naheed Banu

**Affiliations:** ^1^Department of Biochemistry, Faculty of Life Sciences, Aligarh Muslim UniversityAligarh, India; ^2^Department of Biochemistry, Faculty of Medicine & Applied Medical Sciences, Northern Border UniversityArar, Saudi Arabia; ^3^Department of Surgery, Loyola University Medical Center, MaywoodIL, United States; ^4^King Fahd Medical Research Center, King Abdulaziz UniversityJeddah, Saudi Arabia; ^5^Department of Clinical Laboratory Sciences, College of Applied Medical Sciences, King Saud UniversityRiyadh, Saudi Arabia; ^6^College of Medical RehabilitationQassim University, Buraidah, Saudi Arabia; ^7^Novel Global Community Educational Foundation, HebershamNSW, Australia

**Keywords:** *N*-nitrosodiethylamine, chronic unpredictable stress, hepatotoxicity, DNA damage, carcinogenesis

## Abstract

Psychological stress contributes to increased susceptibility to a number of diseases including cancer. The present study was designed to assess the effect of chronic unpredictable stress on *N*-nitrosodiethylamine induced liver toxicity in terms of *in vivo* antioxidant status and DNA damage in Swiss albino mice. The animals used in this study were randomized into different groups based on the treatment with *N*-nitrosodiethylamine or chronic unpredictable stress alone and post-stress administration of *N*-nitrosodiethylamine. The mice were sacrificed after 12 weeks of treatment, and the status of major enzymatic and non-enzymatic antioxidants, liver function markers, lipid peroxidation and the extent of DNA damage were determined in circulation and liver tissues of all the groups. The *N*-nitrosodiethylamine treated group showed significantly compromised levels of the antioxidant enzymes, lipid peroxidation, and the liver function markers with enhanced DNA damage as compared to chronic unpredictable stress or control groups. A similar but less typical pattern observed in the chronic unpredictable stress treated mice. All the measured biochemical parameters were significantly altered in the group treated with the combination of chronic unpredictable stress and *N*-nitrosodiethylamine when compared to controls, or chronic unpredictable stress alone and/or *N*-nitrosodiethylamine alone treated groups. Thus, exposure to continuous, unpredictable stress conditions even in general life may significantly enhance the hepatotoxic potential of *N*-nitrosodiethylamine through an increase in the oxidative stress and DNA damage.

## Introduction

The behavioral processes have long been suspected to influence many health conditions including cancer. Both progression and to a lesser extent cancer onset, have been related to chronic stress, depression, lack of social support and various other psychological factors (Reiche et al., 2004; Antoni et al., 2006). Stress exposure induces a cluster of physiological and behavioral changes that have been linked to the response of the organism to maintain the homeostasis. Moreover, stress has been shown to markedly influence incidence, growth, metastasis, and the rejection of both the chemically induced and implanted tumors (Amkraut and Solomon, 1972; Zygmunt et al., 1985). Both the exacerbation and attenuation of tumor development have, however, been reported in response to stress (Pradhan and Prabhati, 1974; Justice, 1985).

*N*-nitrosodiethylamine (NDEA) causes oxidative stress and cellular injury by enhancing the formation of free radicals (Ramakrishnan et al., 2006; Valko et al., 2006). The metabolic activation of NDEA by cytochrome P450 enzymes converts it to ethyl-acetoxyethyl-nitrosamine, which can be conjugated by the phase II enzymes (Subramanian et al., 2007) to a non-toxic compound. Alternatively, it can form ethyldiazonium ion that directly ethylates cellular macromolecules (Muzio et al., 1999), and is responsible for its cytotoxic, mutagenic, and carcinogenic effects (Abdel-Hamid et al., 2013) through the production of ROS (Bansal et al., 2000).

Oxidative stress, a pervasive condition induced by psychological stress, has been implicated in and recognized to be a prominent feature associated with various diseases like cancer and their progression. An imbalance in the pro-oxidant/antioxidant status indicates the enhanced production of reactive oxygen species (ROS), such as peroxides, hydroxyl, and superoxide anion radicals (Muqbil et al., 2006), which induces cellular oxidative damage through DNA strand breaks and lipid peroxidation (Batcioglu et al., 2002). Thus, DNA damage induced by oxidative stress and/or deficient DNA-repair has an etiological or prognostic role in cancer (Mussarat et al., 1996). A previous study (Ip et al., 1996) has demonstrated the effect of restraint stress on the antioxidant status and DNA damage in rats. It was found that chronic restraint stress increased lipid peroxidation, compromised antioxidant status and enhanced DNA damage (Zaidi et al., 2005; Muqbil and Banu, 2006; Muqbil et al., 2006). Other studies have confirmed that stress is associated with low concentrations of O_6_ methyltransferase, which is an important DNA-repair enzyme (Glaser et al., 1985). Hence, it is important to correlate between alterations in the parameters of psychological stress with those of redox state, DNA-repair activity, and antioxidant defense so as to identify the possible relationship between chronic stress and cancer.

The chronic unpredictable stress (CUS) is a well-recognized model for inducing chronic physical and psychological stress. CUS has been used in the present study to assess the effects of various psychological factors and stressors on some biochemical parameters and DNA damage, also in combination with the known chemical carcinogen NDEA. This study may throw light on the effect of CUS on the early stages of liver cancer as evident from the measured biochemical markers, antioxidant status, and the DNA damage. This also suggests that the life changing unpredictable stresses may enhance the carcinogenic potential of environmental chemicals through compromised oxidative status and DNA damage.

## Materials and Methods

### Chemicals

*N*-nitrosodiethylamine, Histopaque 1077, HBSS, RPMI 1640 were purchased from Sigma, United States (St. Louis, MO), GSH, GSSG, NADPH, NADP, CDNB, DTNB, Pyrogallol were purchased from SRL, India (Muqbil et al., 2006). All other chemicals were of analytical grade purchased from commercial sources. The uric acid (SPN099) and glucose (SPN077) estimation kits were purchased from Span Diagnostics Limited, India.

### Animals

Six-eight week old male Swiss albino mice weighing 35 ± 5 g used for this study were purchased from Jamia Hamdard University, New Delhi, India. The animals were acclimatized for 1 week (Umukoro et al., 2015) and maintained under standard housing conditions with 12 h light and dark cycle. The food in the form of dry pellets (Ashirwad Industries, Chandigarh, India) and water was available *ad libitum*. The Institutional Research Committee approved all animal procedures, and the study has been conducted according to the principles of the University Ethical Committee (UEC) of the Aligarh Muslim University, Aligarh, UP, India, for the Control and Prevention of Cruelty toward Experimental Animals (CPCEA), an Indian National body enacting the ethical rules for performing the research on experimental animals.

### Experimental Design

The experimental mice were randomly divided into four groups of 12 animals each. Group I animals served as normal controls and received no treatments; Group II animals were exposed to CUS for 15 consecutive days just before termination of the experiment. Group III mice were given NDEA (100 mg/kg body weight in normal saline) p.o. once a week for 3 weeks; Group IV animals were treated with NDEA after stress exposure as in group III (post-stress NDEA).

### CUS Procedure

The CUS procedure employed in this study was based on published reports (Katz et al., 1981; Willner et al., 1987; Lu et al., 2006). To maximize unpredictability, different types of stressors were applied in seemingly random order, two per day for 15 days and at various times during the light phase (0800–2000 h) as outlined in **Table 1**.

**Table 1:** Chronic unpredictable stress protocol (Suhail et al., 2011).

Days	Stressor	Time	Days	Stressor	Time
Day 1	1-h restraint stress	08:00 a.m	Day 9	4-hr high-density housing	08:00 a.m
	30 min cold room (4°C)	11:00 p.m		lights on overnight	07:00 p.m
Day 2	1-h shaking/crowding	11:00 a.m	Day 10	4-h wet bedding	09:00 a.m
	5 min cold water swim	03:00 p.m		16-h food and water deprivation	04:00 p.m
Day 3	4-h wet bedding	09:00 a.m	Day 11	3-hr restraint stress	11:00 a.m
	Lights on overnight	06:00 p.m		2-h isolation	05:00 p.m
Day 4	3-hr high-density housing	10:00 a.m	Day 12	5min cold water swim	08:00 a.m
	5 min warm water swim	06:00 p.m		1-h min shaking/crowing	11:00 a.m
Day 5	2-hr restraint stress	08:00 a.m	Day 13	10 min tail pinch in restrainer	09:00 a.m
	30 min cold room (4°C)	03:00 p.m		1-h shaking/crowing	04:00 p.m
Day 6	6-hr isolation	10:00 a.m	Day 14	2-h restraint stress	11:00 a.m
	Lights on overnight	07:00 p.m		3-h high-density housing	04:00 p.m
Day 7	5 min cold water swim	09:00 a.m	Day 15	24-h food and water	08:00 a.m
	16-h food and water deprivation	03:00 p.m		deprivation	
Day 8	2-hr restraint stress	10:00 a.m			
	1-h shaking/crowding	02:00 p.m			


The specific details of the CUS procedure were as follows: for restraint stress, mice were placed individually in body sized wire mesh cages attached to wooden boards, with no movement allowed (Bondi et al., 2008). The shaking-crowding procedure was carried out by placing (Bondi et al., 2008) six mice in a cardboard box atop a lab shaker set to produce 220 back-and-forth movements (Bondi et al., 2008) per minute. Warm swim and cold (Bondi et al., 2008) swim were accomplished by placing the mice in a cylindrical tank (60 cm height-30 cm diameter) filled with water to a 30 cm depth at 25°C or 18°C, respectively. For wet bedding 300 ml tap water was poured in the home cage. The mice were subjected to a high-density housing by placing six mice in a restricted place for 4–6 h. The tail pinch involved placing the mice in the previously described restraining device and applying a clothespin 1 cm from the base of the tail (Suhail et al., 2011) for 10 min.

### Assessment of DNA Damage in Lymphocytes and Liver Cells

Six mice from each group were sacrificed after a period of 12 weeks; their liver and blood tissues were subjected to DNA damage studies by single-cell gel electrophoresis (comet assay). The remaining six mice from each group were sacrificed, and their blood and liver samples were collected and subjected to biochemical estimations.

Immediately after each sacrifice, blood samples of mice were collected in heparinized tubes, and their livers were excised and washed with ice-cold saline. The heparinized blood was suitably diluted in PBS (Ca^2+^ and Mg^2+^ free) (Muqbil et al., 2006). Lymphocytes were isolated using Histopaque 1077 (Sigma), and the cells (≈2 × 10^5^) were finally suspended in RPMI 1640. Excised livers were then filled with Hank’s balanced salt solution (HBSS) Ca^2+^ and Mg^2+^ free, containing proteinase K (17 units/mL) and incubated at 37°C for 30 min. After centrifugation cells were suspended in RPMI 1640. The viability of cells was assessed using trypan blue exclusion test (Pool-Zobel et al., 1993).

The DNA damage in the liver cells was assessed by single alkaline cell gel electrophoresis (comet assay) according to the method of Singh et al. (1988) with slight modification as described earlier (Muqbil and Banu, 2006). The cellular DNA damage was assessed by measuring tail length (migration of DNA from the nucleus in μm) which was automatically generated by Komet 5.5 image analysis system.

### Biochemical Estimations

The biochemical estimations were performed on plasma and liver tissues of mice after 12 weeks of treatment. The plasma of six mice from each group was collected after centrifugation of heparinized blood for 10 min at 2500 × *g* at 4°C. The liver tissues were placed in ice-cold saline containers, and a 10% homogenate was prepared in 0.1 M sodium phosphate buffer, pH 7.4, centrifuged at 10,000 × *g* (4°C) for 15 min to remove cellular debris and the supernatant (Zafir and Banu, 2009) was used for analysis.

The Plasma and liver tissues were used for the estimation of the antioxidant enzymes; superoxide dismutase (SOD), catalase (CAT), glutathione *S*-transferase (GST), and glutathione reductase (GR) as described below. The levels of malondialdehyde (MDA), glutathione (GSH), and liver marker enzymes viz glutamate oxaloacetate transaminase (GOT), glutamate pyruvate transaminase (GPT), and alkaline phosphatase (ALP) were also determined according to the following methods.

#### SOD Activity

Superoxide dismutase activity was assayed by monitoring the inhibition of auto-oxidation of Pyrogallol (0.05 M tris succinate buffer, pH 8.2) at 420 nm (Marklund and Marklund, 1974). One enzyme unit is defined as the amount of enzyme required to cause 50% inhibition of the rate of Pyrogallol auto-oxidation.

#### CAT Activity

The CAT activity was measured in 0.05 M phosphate buffer (pH 7.0) by following the decrease in absorbance at 240 nm due to decomposition of 30 mM hydrogen peroxide (Aebi, 1984; Zafir and Banu, 2009). One enzyme unit is defined as the amount of enzyme (Suhail et al., 2015) decomposing 1 μM H_2_O_2_ per minute at 25°C.

#### GST Activity

The GST activity was assayed in 0.2 M phosphate buffer (pH 6.5) after adding 1 mM 1-chloro-2,4-dinitrobenzene (CDNB) and 1 mM GSH in the reaction mixture and following the increase in absorbance at 340 nm due to the formation of the CDNB–GSH conjugate (Habig et al., 1974). One unit of enzyme activity is defined as the amount of enzyme catalyzing the formation of 1 μM product per min under the specific assay conditions. Enzyme activity was expressed as units per mg of protein (molar extinction coefficient = 9.6 × 10^3^ M/cm).

#### GR Activity

The GR activity was assayed by monitoring the oxidation of 0.1 mM NADPH as a decrease in absorbance at 340 nm (Zafir and Banu, 2009) due to an NADPH-dependent reduction of 1.0 mM oxidized glutathione disulphide to glutathione (0.1 M phosphate buffer, pH 7.6) by the catalytic action of GR (Carlberg and Mannervik, 1975; Zafir and Banu, 2009). One unit of enzyme activity is defined as the amount of enzyme catalyzing the 1 μM NADPH per min under assay conditions. Enzyme activity was calculated using a molar extinction coefficient of 6.22 × 10^3^ M/cm and expressed as units per mg of protein.

#### Total Reduced Glutathione

The levels of GSH were determined using the classical thiol reagent 5,5′-dithiobis-2-nitrobenzoic acid (DTNB) (0.1 M phosphate buffer, pH 7.4). The yellow color developed by the reaction of GSH with DTNB was read at 412 nm (Jollow et al., 1974).

#### Uric Acid and Glucose

The levels of Uric Acid and Glucose in plasma were measured by using commercial kits (Span Diagnostics Ltd., India).

#### Lipid Peroxidation, Malondialdehyde (MDA)

The lipid peroxidation was measured by determining MDA (a thiobarbituric acid reactive species, TBARS) spectrophotometrically following the thiobarbituric acid (TBA) test for the formation of TBARS during an acid-heating (Bondi et al., 2008) reaction (Beuge and Aust, 1978). The pink chromogen formed by MDA–TBA complex was detected at 535 nm and quantified using an extinction coefficient of 1.56 × 10^3^ M/cm (Zafir and Banu, 2009).

#### Liver Marker Enzymes

The commercial kits (Span Diagnostics Ltd., India) were used for the measurements of the GOT and GPT in the liver tissues and plasma. The ALP activity was measured in the circulation and liver tissues by using p-nitro phenylphosphate (pNPP) as substrate (Shah et al., 1979).

#### Protein Estimation

The protein content was determined by the method of Lowry et al. (1951) using bovine serum albumin as standard.

#### Statistical Analysis

The data were expressed as group mean ± SEM of six values and analyzed by one-way ANOVA for differences among controls and treatment groups. The ‘*p*’ values of 0.05 or less were considered statistically significant.

## Results

### Effect of CUS and NDEA on Lipid Peroxidation and GSH Levels

**FIGURE 1:**
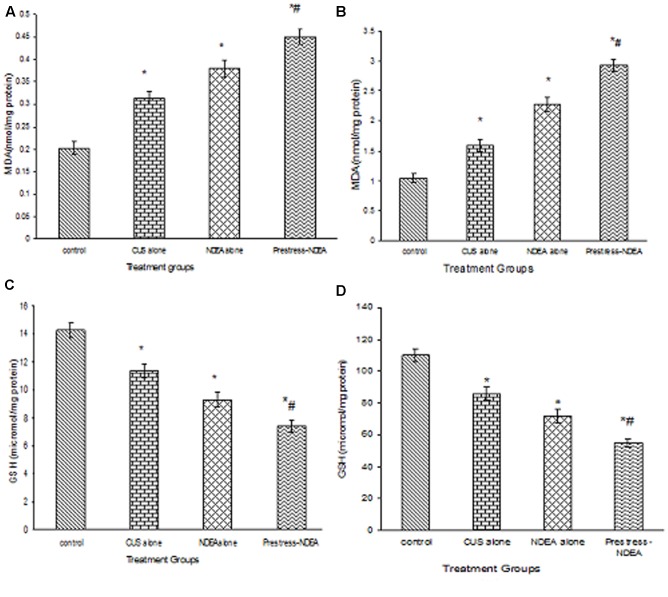
The effect of chronic unpredictable stress (CUS) and *N*-nitrosodiethylamine (NDEA), alone and in combination on the plasma **(A)**, hepatic **(B)** levels of malondialdehyde (MDA); and plasma **(C)** and hepatic **(D)** levels of GSH in the experimental mice. ^∗^*P* < 0.05 when compared to control group. ^#^*P* < 0.02 when compared to CUS alone and NDEA alone treated.

The plasma and liver tissue levels of MDA showed a significant (*p* < 0.05) increase when the mice were exposed to CUS or NDEA alone as compared to controls. The post-stress NDEA administration further significantly (*P* < 0.02) enhanced the levels as compared to all the other groups (**Figures 1A,B**). While the levels of GSH were significantly (*P* < 0.05) decreased in the plasma and liver tissues of CUS alone or NDEA alone treated mice, as compared to control groups, they were further decreased significantly (*P* < 0.02) in the post-NDEA stress treated mice as compared to either CUS or NDEA alone treated groups (**Figures 1C,D**).

### Effect of Various Treatments on the Antioxidant Status

The activities of antioxidant enzymes, SOD, CAT, GST, and GR in liver tissues were significantly (*P* < 0.05) decreased in CUS alone, and NDEA alone treated groups, as compared to the controls (**Table 2**). The activities were further significantly (*P* < 0.02) decreased in post-NDEA stress treated mice as compared to the other groups.

**Table 2:** The activities of free radical metabolizing enzymes in the liver tissues of mice exposed to chronic unpredictable stress (CUS) alone, *N*-nitrosodiethylamine (NDEA) alone, and post-stress NDEA treatments.

Groups	SOD units/mg protein	CAT units/mg protein	GST units/mg protein	GR units/mg protein
Control	553.9 ± 8.4	27.3 ± 1.4	594.1 ± 9.6	0.277 ± 0.01
CUS alone	399.3^∗^ ± 7.7	18.6^∗^ ± 1.2	424.5^∗^ ± 9.2	0.125^∗^ ± 0.004
NDEA alone	275.4^∗^ ± 8.5	15.2^∗^ ± 0.8	329.2^∗^ ± 9.5	0.082^∗^ ± 0.005
Post-stress NDEA	227.4^∗#^± 6.2	9.1^∗#^± 0.43	219.6^∗#^± 9.1	0.045^∗#^± 0.003


*Each value represents the mean ± SEM of six animals. ^∗^*P* < 0.05 when compared to control group. ^#^*P* < 0.02 when compared to CUS alone and NDEA alone treated groups.*

### Alterations in the Functional Markers and Biochemical Parameters by CUS and NDEA Exposure Alone or Combination

The levels of various marker enzymes of liver function (Muqbil et al., 2006) were significantly (*p* < 0.05) increased in the liver tissues of stressed as well as NDEA alone treated mice (**Table 3**). The levels were further increased significantly (*p* < 0.02) in post-NDEA stress treated mice as compared to either control, stress alone or NDEA alone treated groups. The circulating levels of the biochemical liver markers of control and treated groups showed a similar pattern. The *in vivo* antioxidant levels were significantly (*p* < 0.05) decreased in CUS alone, and NDEA alone treated groups as compared to controls (**Table 4**). These activities were further significantly (*p* < 0.02) decreased in the post-NDEA stress treated mice as compared to other groups. In the CUS alone and NDEA alone treated groups the circulatory levels of uric acid and glucose were significantly decreased (*p* < 0.05) as compared to controls, however, these levels were further significantly decreased (*p* < 0.02) in post-NDEA stress treated group as compared to NDEA alone and/or CUS alone treatments (**Table 4**).

**Table 3:** The tissue levels of liver function enzymes GOT, GPT, and ALP in the mice exposed to CUS alone, NDEA alone, and post-stress NDEA treatments.

Groups	GOT IU/L	GPT IU/L	ALP mg/ml
Control	46.4 ± 2.1	28.7 ± 1.3	8.8 ± 0.5
CUS alone	60.2^∗^ ± 2.9	40.1^∗^ ± 2.8	13.8^∗^ ± 0.5
NDEA alone	83.6^∗^ ± 2.2	95.7^∗^ ± 1.5	19.6^∗^ ± 1.1
Post-stress NDEA	101.1^∗#^± 1.3	129.2^∗#^± 2.3	34.4^∗#^± 1.0


*Each value represents the mean ± SEM of six animals. ^∗^*P* < 0.05 when compared to control group. ^#^*P* < 0.02 when compared to CUS alone and NDEA alone treated groups.*

**Table 4:** The effect of CUS alone, NDEA alone, and post-stress NDEA treatments on various circulatory biochemical parameters.

	SGOT	SGPT	ALP	Glucose	Uric acid	SOD units/mg	GST units/mg
Groups	IU/L	IU/L	mg/ml	mg/100 ml	mg/100 ml	protein	protein
Control	15.42 ± 0.51	24.13 ± 1.22	5.60 ± 0.31	110.20 ± 4.33	5.84 ± 0.32	40.78 ± 1.83	0.75 ± 0.03
CUS alone	23.10^∗^ ± 1.11	36.31^∗^ ± 1.50	7.93^∗^ ± 0.42	85.53^∗^ ± 3.52	2.99^∗^ ± 0.12	31.20**^∗^** ± 1.3	0.43^∗^ ± 0.02
NDEA alone	57.81^∗^ ± 2.62	59.43^∗^ ± 2.21	14.51^∗^ ± 0.41	70.62^∗^ ± 3.51	2.10^∗^ ± 0.11	23.29^∗^ ± 1.33	0.30^∗^ ± 0.01
Post-stress NDEA	74.53^∗#^± 2.10	89.32^∗#^ ± 1.72	25.52^∗#^± 0.86	53.91^∗#^ ± 1.50	0.93^∗#^± 0.05	14.78^∗#^± 0.57	0.20^∗∗^ ± 0.02


*Each value represents the mean ± SEM of six animals. ^∗^*P* < 0.05 when compared to control group. ^#^*P* < 0.02 when compared to CUS alone and NDEA alone treated groups. ^∗∗^*p* < 0.01 as compared to the controls.*

### The Effect of Various Treatments on the Lymphocyte and Liver Cell DNA Damage

**FIGURE 2:**
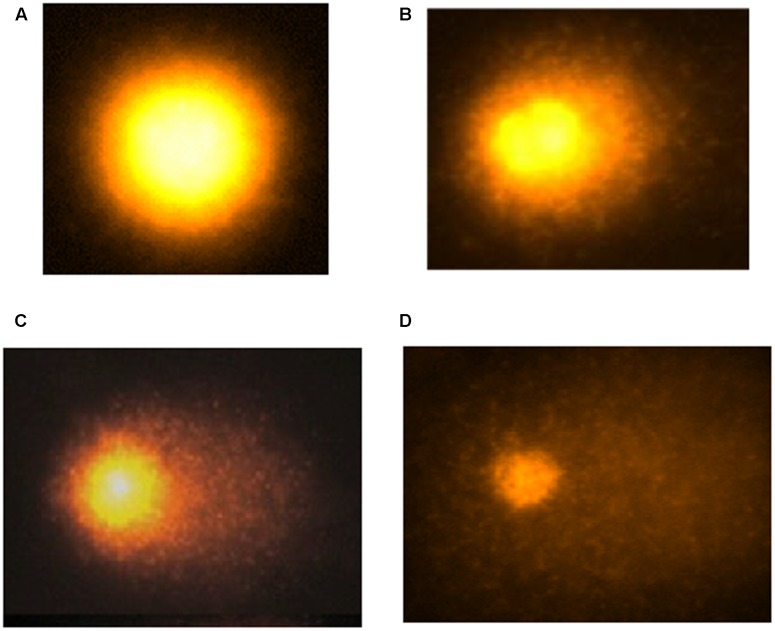
The hepatocyte DNA damage measured as comet tail lengths in control **(A)** and after CUS **(B)**; NDEA **(C)**; and post-CUS NDEA **(D)** treatments in the mice.

Both in the lymphocytes and liver cells, the DNA damage was increased significantly (*p* < 0.05) both in CUS or NDEA alone treated mice as compared to the control groups as depicted by comet tail lengths (**Table 5**). The DNA damage was further increased significantly (*p* < 0.02) by post-stress NDEA treatment as compared to the other groups (**Table 5**; liver cells **Figure 2**).

**Table 5:** The DNA damage caused by CUS, NDEA, and post-stress NDEA treatments on the peripheral blood lymphocytes and the liver cells of mice.

		CUS	NDEA	Post-stress
Groups	Control	alone	alone	NDEA
Lymphocyte Comet				
tail length (μm)	2.16 ± 0.41	10.24^∗^ ± 1.31	30.8 ± 0.75	40.19^∗#^ ± 1.43
Liver cell Comet				
tail length (μm)	2.37 ± 0.33	12.90^∗^ ± 0.75	44.12^∗^ ± 0.88	54.71^∗#^ ± 1.01


*Data represent means ± SEM of six animals in each group. ^∗^*P* < 0.05 when compared to control group. ^#^*P* < 0.02, when compared to CUS alone and NDEA alone, treated groups.*

Thus, these results reveal that the CUS treatment aggravates the NDEA induced alterations of lipid peroxidation, antioxidant status, and biochemical markers, leading to an increase in carcinogen induced oxidative stress and DNA damage, which may play a significant role in enhancing the carcinogenic potential of NDEA in liver carcinogenesis.

## Discussion

Exposure to stress situation stimulates numerous pathways leading to increased production of free radicals. Several human chronic diseases including cancer have been associated with the altered oxidative stress, produced either through an increased free radical generation and/or a compromised antioxidant level in the target cells and tissues (Trush and Kensler, 1991; Rice-Evans and Burdon, 1993). The free radicals thus generated induce strand breaks and cause oxidative modification of DNA bases (Cerutti, 1985). The participation of ROS in hepatocarcinogenesis is evident as initiation with low doses of NDEA induces liver DNA-8-hydroxydeoxy-guanosine adduct formation (Nakae et al., 1997) and causes mutations, which leads to carcinogenesis. The free radicals, mostly ROS cause cellular injury. The consequences of which are often exhibited and measured as lipid peroxidation product MDA, the major end product of this reaction. Malondialdehyde directly reacts with DNA to form oxidative DNA adducts (Chaudhary et al., 1994), thereby causing DNA damage and interfering with the DNA-repair mechanism.

The present study shows that exposure of mice to CUS result in a significant increase in the levels of MDA, and a significant decrease in the antioxidant enzyme activities and the levels of GSH. These results are supported by previous studies in which CUS showed a disturbed oxidant-antioxidant balance (Zhang et al., 2009). The significant increase of MDA in NDEA-administered mice has also been reported by others (Thirunavukkarasu and Sakthisekaran, 2001; Pradeep et al., 2007). However, the levels of MDA were further significantly increased in post-NDEA stress treated group, which could be due to either increased production or decreased destruction of ROS, as the exposure to stress already causes a compromised status of the antioxidant system and thus decrease in anti-oxygenic potential *in vivo*. Reduced GSH is the major cytosolic thiol compound which plays important cellular functions including the destruction of hydrogen peroxide, lipid peroxides, and free radicals. The administration of NDEA caused a significant decrease in the levels of GSH, which may be responsible for the increased lipid peroxidation. NDEA administration after stress exposure caused a further decrease in the levels of GSH. This enhanced decrease of GSH in post-stress NDEA treatment suggests that the cells which are already deficient in thiol (-SH) groups undergo fast lipid per oxidation, as GSH is one of the guarding factors against oxidative stress. Enhanced lipid peroxidation associated with depletion of antioxidants is a characteristic finding in a variety of malignancies (Anis et al., 2001). The primary antioxidant enzymes SOD and CAT are involved in the inactivation of environmental carcinogens and direct elimination of toxic free radicals and electrophiles *in vivo*, which helps in the amelioration of the oxidative damage. The activities of both these free radical scavenging enzymes were found to be decreased in rats treated with NDEA (Yadav and Bhatnagar, 2007). The results of our study also indicate that the NDEA administration increases the susceptibility of hepatocytes to carcinogenesis by reducing the activities of SOD and CAT. The oxidative stress through the generation of ROS causes denaturation of protein/enzyme by the formation of protein carbonyls which might have caused inhibition of enzymatic activities (Höhn and Grune, 2014). A further significant decrease in the activities of SOD and CAT by CUS in NDEA-treated mice could be due to over utilization/less production of these enzymatic antioxidants to scavenge the products of lipid peroxidation and oxidative stress.

A large number of studies have established a correlation between cancer incidence and various disorders of GSH-related enzyme functions, alterations of GSTs being most frequently reported. A significant decrease in the activity of GSH dependent enzymes, GST and GR was observed in NDEA treated mice. This may be due to the decreased expression of these antioxidants during hepatocellular damage as previously described (Kweon et al., 2003). The further decreased levels of GST and GR as observed in the post-stress NDEA treated mice might have facilitated the NDEA induced liver carcinogenesis by decreasing its clearance from the system. The already compromised state of antioxidant level due to stress exposure might have further contributed in aggravation of the oxidative stress and NDEA induced toxicity. The diagnostic biomarker enzymes of hepatic damage GOT, GPT, and ALP are released into the circulation after cellular damage (Naik and Panda, 2007) and a rise of these marker enzymes in the serum causes increased permeability and necrosis of the cells (Al-Athar, 2004). In this study, we demonstrated that the administration of NDEA to mice leads to a marked elevation in the levels of GOT, GPT, and ALP which is indicative of hepatocellular damage, as also previously reported (Sadik et al., 2008). Further elevations of these enzymes in post-stress NDEA treatment could potentially be attributed to the release of these enzymes from the cytoplasm into the blood (Ritter et al., 2004) circulation after rupture of the plasma membrane due to increased free radical induced cellular damage. Both uric acid and glucose are also considered to be the major antioxidants found in blood plasma. The uric acid can act as an antioxidant either by binding to the radicals or by directly scavenging oxidizing species. It, therefore, inhibits lipid peroxidation (Muqbil et al., 2006). Glucose also acts as a scavenger of hydroxyl radicals (Halliwel and Gutteridge, 1990). The levels of these antioxidants were found to be decreased with increase in lipid peroxidation in plasma of mice exposed either to CUS alone or NDEA alone. The levels were further decreased when NDEA was given to the mice after exposure to stress, which shows that stress itself alters the antioxidant status, thus facilitating (Muqbil et al., 2006) and enhancing the NDEA induced liver carcinogenesis. Earlier studies from our laboratory have shown that CUS enhanced both the nephrotoxic and hepatotoxic potential of 7,12-dimethylbenz(a) anthracene in Swiss albino mice (Suhail et al., 2011), by altering the oxidative stress and compromising the antioxidant system, thus stress may act as a promoter of carcinogenesis by enhancing the pro-oxidant potential of carcinogens. Moreover, CUS also aggravated the development of skin tumors (in terms of their incidence, tumor yield, and tumor burden) in mice which further strongly supported our studies on the effect of stress on cancer promotion through DNA damage and modulation of oxidant/antioxidant system *in vivo* (Suhail et al., 2015). Thus, irrespective of the carcinogen used or the mode of application topical (Suhail et al., 2011, 2015) or oral as in the present study, CUS increased the toxic potential of the carcinogens.

All the above findings of oxidative stress were also supported by studies which showed CUS and NDEA caused significant damage to the DNA of lymphocytes and the liver cells as compared to controls. Post-CUS NDEA treatment showed further higher DNA damage in comparison with control, CUS alone or NDEA alone groups. The explanation for this may be that the chronic stress caused DNA damage within the cell either by altering the ability of the cells to repair DNA due to compromised antioxidant defense system (Bondi et al., 2008), or by causing oxidative stress and inhibiting apoptosis as observed by others also (Glaser et al., 1985; Kiecolt-Glaser et al., 1985; Adachi et al., 1993). The mechanisms that play roles in NDEA carcinogenicity in hepatocytes include DNA adduct formation followed by a gene mutation (Dyroff et al., 1986; Deal et al., 1989; Travis et al., 1991). Administration of NDEA generates lipid peroxidation products in general (Hietanen et al., 1987) and enhances the formation of the activated oxygen species in the preneoplastic nodules (Scholz et al., 1990) in the liver. The increased formation of reactive oxygen substances both in the circulation and in the liver tissues by NDEA administration either alone or after stress exposure further confirms an enhanced DNA damage by the chronic stress. Thus, the chronic stress plays an important role in the initial stages of liver carcinogenesis by compromising the antioxidant status, inducing oxidative stress and enhancing the DNA damaging potential of a carcinogen. Human body remains in a compromised antioxidant and enhanced oxidative status due to physical, physiological or psychological stress, and under this condition, any insult due to environmental carcinogens can aggravate the situation and may pave the way for carcinogenesis by enhancing the carcinogenic potential of any potent carcinogen irrespective of the mode of exposure.

## Conclusion

Our results suggest a strong correlation between the observed alterations in the biochemical parameters, notably decreased antioxidant status and increased oxidative stress, due to the chronic stress exposure and the toxicity of environmental chemicals. Exposure to NDEA caused induction of oxidative stress and DNA damage by the generation and accumulation of large amounts of free radicals. Chronic stress further exacerbated this condition, thus putting at an increased risk of developing cancer. This study may offer a sensitive and useful approach for assessment of the effects of psychological stress on the carcinogenic risks of environmental chemicals (Bondi et al., 2008). Further studies may be aimed to combine the parameters of psychological stress with those of redox state, DNA-repair activity, and antioxidant defense system, so as to identify the relationship between psychological/physical stress and carcinogenesis at the molecular level to give a clearer picture.

## Ethics Statement

This study was carried out after the protocol was approved by the Aligarh Muslim University ethics committee in accordance with the recommendations of the Indian national committee for the ‘Control and Prevention of Cruelty towards Experimental Animals’.

## Author Contributions

NB conceived and designed the study. NB, NS, GA, HK, and SH performed the experiments and wrote the manuscript. NB, AA, MA, and SF provided critical revision of the manuscript for intellectual content.

## Conflict of Interest Statement

The authors declare that the research was conducted in the absence of any commercial or financial relationships that could be construed as a potential conflict of interest.
